# JAK/STAT guarantees robust neural stem cell differentiation by shutting off biological noise

**DOI:** 10.1038/s41598-018-30929-1

**Published:** 2018-08-20

**Authors:** Yoshitaro Tanaka, Tetsuo Yasugi, Masaharu Nagayama, Makoto Sato, Shin-Ichiro Ei

**Affiliations:** 1 Department of Mathematics, Faculty of Science, Hokkaido University, Kita 10, Nishi 8, Kita-Ku, Sapporo, Hokkaido 060-0810 Japan; 2 Mathematical Neuroscience Unit, Institute for Frontier Science Initiative, Kanazawa University, 13-1 Takaramachi, Kanazawa-shi, Ishikawa 920-8640 Japan; 3 Research Institute for Electronic Science, Hokkaido University, Kita 12, Nishi 7, Kita-ku, Sapporo, Hokkaido 060-0812 Japan; 4 Laboratory of Developmental Neurobiology, Graduate School of Medical Sciences, Kanazawa University, 13-1 Takaramachi, Kanazawa-shi, Ishikawa 920-8640 Japan; 5 Present Address: Department of Complex and Intelligent Systems, School of Systems Information Science, Future University Hakodate, 116-2 Kamedanakano-cho, Hakodate, Hokkaido 041-8655 Japan

## Abstract

Organismal development is precisely regulated by a sequence of gene functions even in the presence of biological noise. However, it is difficult to evaluate the effect of noise *in vivo*, and the mechanisms by which noise is filtered during development are largely unknown. To identify the noise-canceling mechanism, we used the fly visual system, in which the timing of differentiation of neural stem cells is spatio-temporally ordered. Our mathematical model predicts that JAK/STAT signaling contributes to noise canceling to guarantee the robust progression of the differentiation wave *in silico*. We further demonstrate that the suppression of JAK/STAT signaling causes stochastic and ectopic neural stem cell differentiation *in vivo*, suggesting an evolutionarily conserved function of JAK/STAT to regulate the robustness of stem cell differentiation.

## Introduction

During development, stochastic noise arises to varying degrees. Stochastic gene expression and fluctuation of proteins occur intrinsically, caused by the probabilistic collisions between molecules. In addition, cell divisions and external forces from the environment often affect cell positioning and cell fate decisions. Any individual multicellular organism can robustly develop even under these intrinsic and extrinsic sources of noise. Therefore, noise resistance is one of the essential properties of the developing multicellular organisms^1^. For robust gene expression in a cell, negative feedback is one of the major ways to reduce the effects of stochastic noise^2,3^. Similarly, the ratio of transcription frequency to translation frequency attenuates intrinsic noise^4^. It has been reported that intercellular Notch signaling plays the crucial role to minimize the noise for consistent coherent oscillation during somite segmentation^5^. However, the specific mechanisms of the noise resistance in developing multicellular systems have rarely been identified.

Mathematical modeling is a useful tool for estimating the effects of stochastic noises and fluctuations in biological systems. For unicellular organisms, stochastic models can be used to control and regulate the stochastic gene expression levels and the fluctuation of protein expression^2,6,7^. These models are usually described as simple ordinary differential equations (ODEs) by assuming that the mutual interaction among networks is linear. For multicellular organisms, it is necessary to construct the mathematical model based on partial differential equations (PDEs) and/or the ODEs with intercellular interactions because intercellular signaling plays significant roles in the development of multicellular organisms. By varying the values of the parameters in the mathematical models, we can observe the behaviors of the solutions corresponding to the biological phenomena, and we can easily add small amounts of noise to the specified regions in the numerical simulations. Moreover, if we use simple biological models such as the fruit fly, we can adapt the parameters of the mathematical models to the real genes in the network systems, and we can freely manipulate them in the numerical simulations. Thus, by changing the parameters in the numerical simulations in the presence of noise and observing the numerical results of the mathematical model, we can directly identify the gene network relating to the robustness against the biological noises.

**Figure 1:**
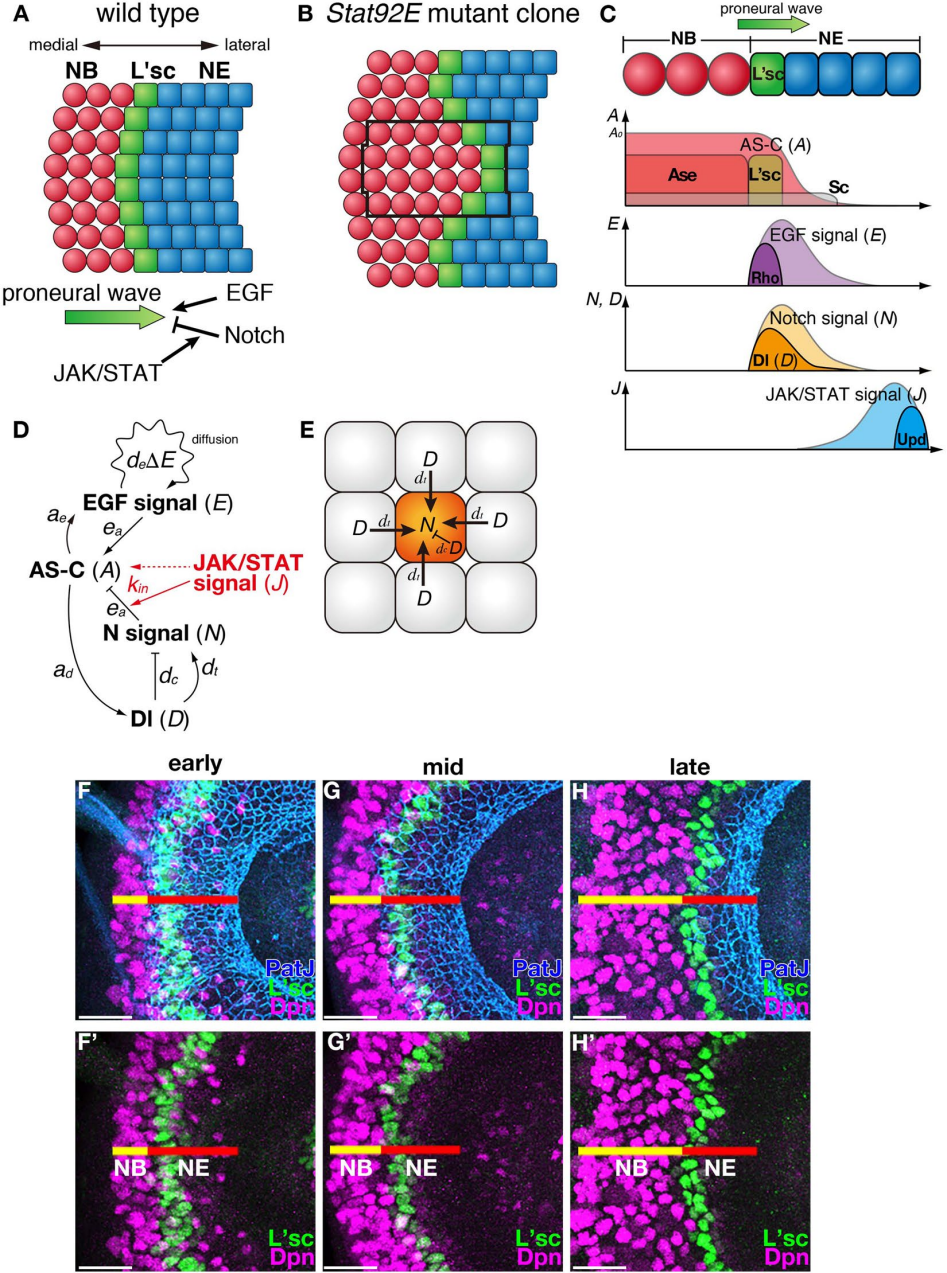
The proneural wave progresses unidirectionally during optic lobe development in *Drosophila*. (**A**) A schematic of the progression of the proneural wave. The proneural wave sweeps from medial to lateral. L’sc is transiently expressed in the differentiating NEs and defines the timing of the NE-to-NB differentiation. EGF positively regulates the progression of the wave, while Notch and JAK/STAT negatively regulate wave progression through increasing the expression of Notch target genes. (**B**) Summary of the loss-of-function phenotype of the *Stat92E* mutant clone. (**C**) Schema showing the spatial profile of each signaling activity. (**D**) Schema showing the gene regulatory network including AS-C, EGF, Notch, Dl, and JAK/STAT. (**E**) Lateral inhibition mechanism of Delta/Notch signaling. (**F**–**H’**) The progression of the proneural wave *in vivo*. Control third-instar larvae at (**F**) 114-126, (**G**) 126–138, (**H**) 138–150 hours after egg laying are shown. PatJ (blue, NE), L’sc (green, proneural wave), Deadpan (Dpn, magenta, NB) are shown. (**F’**, **G’**, and **H’**) L’sc (green) and Dpn (maenta) are shown. Scale bars, 20 μm.

To theoretically and experimentally investigate how multicellular organisms cope with biological noise, the developing fly visual system provides an excellent model. The wave of differentiation called the “proneural wave” sweeps across the surface of the brain during the third larval instar (Fig. 1A)^8,9^. During the progression of the proneural wave, neuroepithelial cells (NEs) sequentially differentiate into neuroblasts (NBs), neural stem-like cells. This progression starts at the medial edge of the NE sheet and extends toward the lateral edge. The differentiating cells start expressing Achaete-Scute Complex (AS-C) proneural proteins such as Scute, Lethal of Scute (L’sc), and Asense (Ase)^8^. Among them, L’sc is transiently expressed in cells at the transition interface between NEs and NBs and acts as a trigger for NB differentiation (Fig. 1A,F–H’)^8^. During the progression of the proneural wave, several signaling pathways, including EGF, Notch, JAK/STAT, and Hippo, play pivotal roles^8–12^. The EGF and Notch signaling pathways are activated in the wavefront cells and regulate the progression of the wave positively and negatively, respectively (Fig. 1A,C)^9,12^. By combining mathematical modeling with genetic experiments, we have previously reported that Notch-mediated lateral inhibition is implemented within the progression of the proneural wave, though Notch activation does not show a salt and pepper pattern^13^.

In this study, we show that our previous mathematical model lacks noise resistance. This raises a possibility that there is a noise-canceling mechanism during the progression of the proneural wave *in vivo*. To improve our previous model, we focus on the function of JAK/STAT signaling, which has been shown to negatively regulate the progression of the wave *in vivo*^8^. The modified version of the mathematical model cancels the effect of noise and shows the robust progression of the wave. We further demonstrate that suppression of the function of *Stat9*2*E* shows ectopic and random NB differentiation *in vivo*. Our *in silico* and *in vivo* data indicate that JAK/STAT signaling plays a critical role in noise canceling during neural stem cell differentiation.

## Results

### Mild EGF noise induces ectopic neuroblast differentiation in the previous mathematical model

To understand the complex interplay among EGF, Notch, and AS-C during the propagation of the proneural wave, we have previously developed a mathematical model based on the molecular interactions identified through a series of genetic experiments (Fig. 1D,E)^13^. We have applied the diffusion model for EGF and the lateral inhibition mechanism for Notch and have represented the mutual interactions as follows:

1 $$\begin{array}{c}(Model\,\,\,1)\{\begin{array}{ccc}\frac{{\rm{\partial }}E}{{\rm{\partial }}t} & = & {d}_{e}{\rm{\Delta }}E-{k}_{e}E+{a}_{e}A({A}_{0}-A),\\ \frac{d{N}_{i,j}}{dt} & = & -{k}_{n}{N}_{i,j}+{d}_{t}\sum _{l,m\in {{\rm{\Lambda }}}_{i,j}}{D}_{l,m}-{d}_{c}{N}_{i,j}{D}_{i,j},\\ \frac{d{D}_{i,j}}{dt} & = & -{k}_{d}{D}_{i,j}+{a}_{d}{A}_{i,j}({A}_{0}-{A}_{i,j}),\\ \frac{d{A}_{i,j}}{dt} & = & {e}_{a}({A}_{0}-{A}_{i,j})max\{{E}_{i,j}-{N}_{i,j},0\}.\end{array}(x,y)\in B,\,t > 0,\end{array}$$

Here, *E* is a composite variable for the EGF ligand concentration and EGF signaling at position *x* and time *t*, *N*_*i*,*j*_ and *D*_*i*,*j*_ are variables for Notch signal activity and Dl expression in the *i*th and *j*th cells at time *t*, respectively, *A*_*i*, *j*_ is a variable for the level of the differentiation of AS-C in the *i*th and *j*th cells at time *t*, and *d*_*e*_, *k*_*e*_, *a*_*e*_, *k*_*n*_, *d*_*t*_, *d*_*c*_, *k*_*d*_, *a*_*d*_, *e*_*a*_ are positive constants (see materials and methods for other definitions). This suggests that the combination of the reaction diffusion system of EGF and the lateral inhibition system of Notch accurately mimics *in vivo* situations in wild-type animals and mutants for EGF or Notch signaling components^13^.

**Figure 2:**
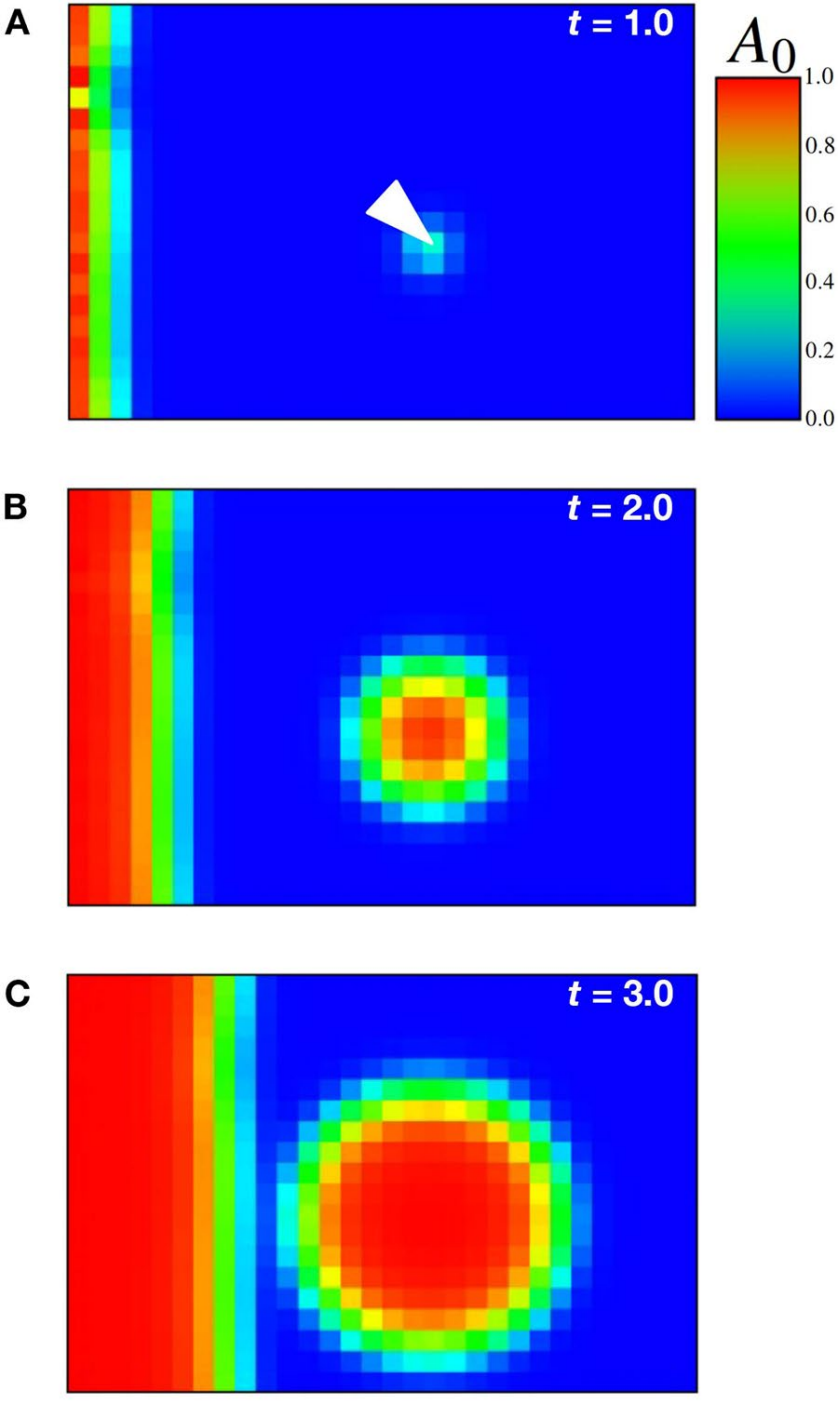
Minor perturbation of EGF signaling away from the wavefront causes spontaneous NB differentiation in Model 1. The small perturbation is imposed on *E* at the white arrow in the initial condition as shown in (**A**). The parameters are *d*_*e*_ = 2.0, *k*_*e*_ = 1.0, a_e_ = 4.0, *k*_*n*_ = 3.0, *d*_*t*_ = 2.0, *d*_*c*_ = 0.1, *k*_*d*_ = 3.0, *a*_*d*_ = 4.0, *e*_*a*_* = *10.0 and *A*_0_ = 1.0. Panels (**A**–**C**) correspond to the profiles of *A* at *t* = 1.0, *t* = 2.0, and *t* = 3.0, respectively. The high and low expression levels of *A* are shown in red and blue as indicated in the color bar.

Although Model 1 reproduces experimental results for the progression of the proneural wave, it lacks the noise resistance. The addition of noise for EGF in the numerical simulation causes stochastic NB differentiation apart from the proneural wavefront (Fig. 2). The numerical results of Model 1 with the addition of a small amount perturbation of the EGF level away from the wavefront at the white arrow shows the propagation of spontaneous NB differentiation in the region distant from the wavefront. In contrast, the effect of artificial activation of EGF signaling ahead of the wavefront is very limited *in vivo*. Although ectopic NB differentiation is induced, it does not propagate to the surrounding undifferentiated region^12^. The difference between *in silico* and *in vivo* experiments suggests that there is a noise-canceling mechanism to ensure the robust progression of the differentiation wave *in vivo*.

### JAK/STAT signaling suppresses the effect of noise during the progression of the proneural wave *in silico*

As a candidate mechanism for the noise canceling, we focused on the function of JAK/STAT signaling based on previous findings. First, Unpaired (Upd), a ligand for JAK/STAT signaling, is expressed in lateral NEs and forms a gradient of JAK/STAT signaling, which is higher laterally and lower medially (Fig. 1C, Fig. S1)^8^. Second, JAK/STAT signaling negatively regulates the progression of the proneural wave; therefore, suppression of JAK/STAT signaling results in faster progression of the proneural wave (Fig. 1B)^8^. Third, JAK/STAT signaling positively regulates the expression of a subset of Notch target genes, including *Enhancer of split mδ* (*E(spl)m*δ), *Enhancer of split m7* (*E(spl)m7*), and *Twin of m4* (*Tom*), in the fly brain^14^. We therefore improved Model 1 by incorporating the effect of JAK/STAT into the system. Based on previous findings, we added the following assumptions to construct the mathematical models with noise resistance:

(A.1) JAK/STAT activates the expression of a subset of Notch target genes that inhibit the expression of the AS-C (Fig. 1D).

(A.2) JAK/STAT signaling forms a gradient under the control of Upd and reaches the stationary state independently of the propagation of the proneural wave.

**Figure 3:**
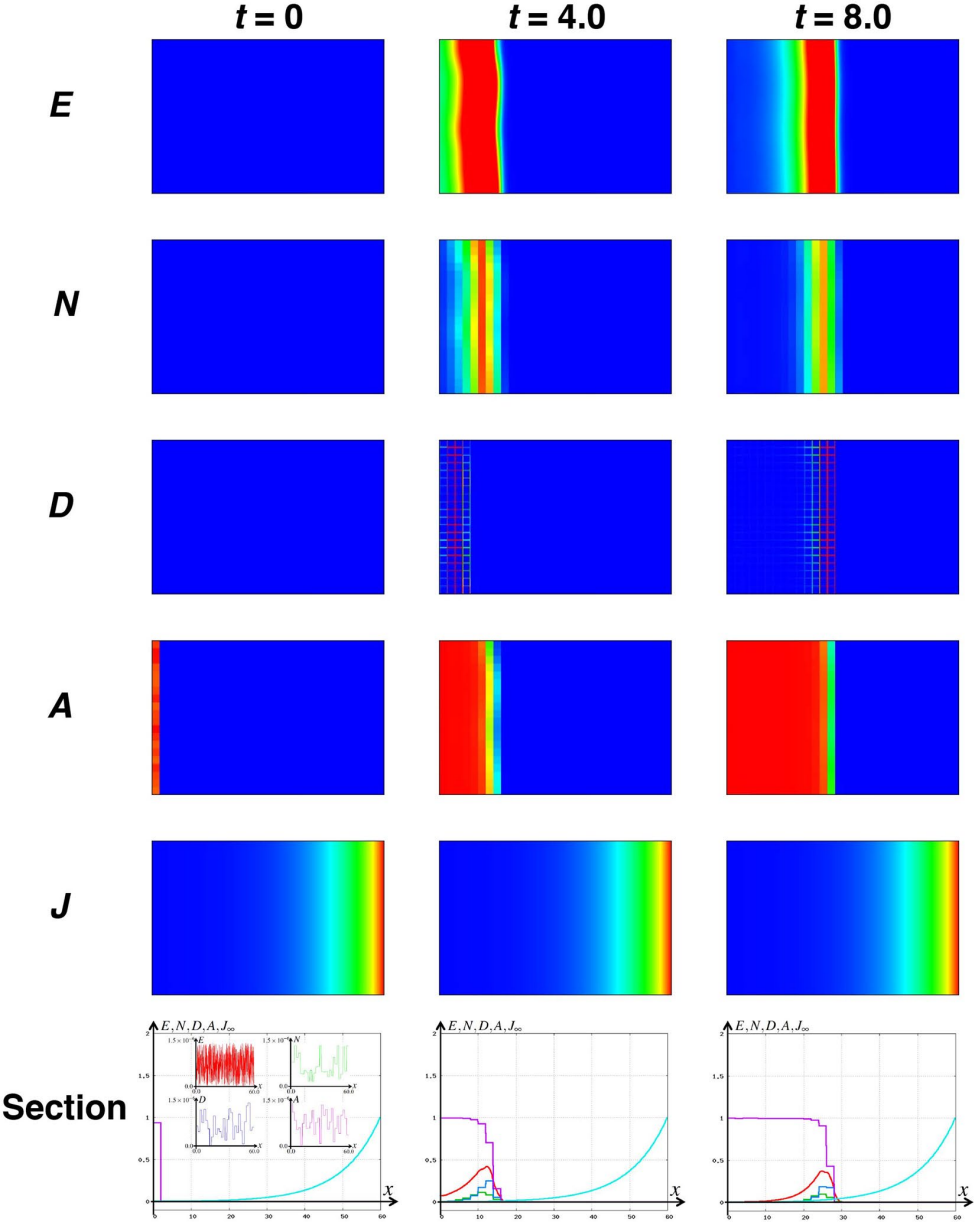
JAK/STAT signaling cancels the noise *in silico*. The result of the numerical simulation of Model 2 in the field *B* with the same parameters as in Fig. 2 and *k*_*in*_ = 1.0, *d*_*i*_ = 1.0, *k*_*i*_ = 0.01, *J*_0_ = 1.0. The five upper figures show the profiles of the *E*, *N*, *D*, *A*, and *J* in the field *B*, and the lowest panels show the longitudinal section, where red, green, blue, magenta, and light blue curves correspond to the values of *E*, *N*, *D*, *A*, and *J*_∞_ on [0, *L*_*x*_] and *y* = *L*_*y*_*/2*. The columns from left to right show the profiles of *t* = 0, *t* = 4.0, and *t* = 8.0, respectively. The insets in the longitudinal section at *t* = 0 are enlarged views of *E*, *N*, *D*, and *A* at *t* = 0, and similar noise is added in each step in the numerical simulation.

In Model 1, the effect of Notch corresponding to the *E(spl)m*δ and *E(spl)m7* is imposed inside the max function of the equation of *A*. This term regulates the trigger of the differentiation of *A*. Because JAK/STAT regulates these Notch target genes, we modified the fourth equation of Model 1 according to the assumption (A.1):

2 $$\frac{d{A}_{i,j}}{dt}={e}_{a}({A}_{0}-{A}_{i,j})max\{{E}_{i,j}-{N}_{i,j}-{k}_{in}{J}_{{\rm{\infty }}},0\},$$

where *J* is denoted by the JAK/STAT signaling at position *x* and time *t* and *k*_*in*_ is a positive constant for the regulation rate of JAK/STAT signaling for Notch target genes. Furthermore, from the assumption (A.2), as the JAK/STAT creates the gradient of its activation strongly in the lateral region and weakly in the medial region (Fig. S1), we impose *J*_∞_ as a stationary state for the following reaction-diffusion model:

3 $$\{\begin{array}{ccc}\frac{{\rm{\partial }}J}{{\rm{\partial }}t} & = & {d}_{i}{\rm{\Delta }}J-{k}_{i}J,\,(x,y)\in B,\,\,t > 0,\\ \frac{{\rm{\partial }}J}{{\rm{\partial }}y}(t,x,0) & = & \frac{{\rm{\partial }}J}{{\rm{\partial }}y}(t,x,{L}_{y})=0,\,x\in [0,{L}_{x}],\\ \frac{{\rm{\partial }}J}{{\rm{\partial }}x}(t,0,y) & = & 0,\,J(t,{L}_{x},y)={J}_{0},\,y\in [0,{L}_{y}],\end{array}$$

where *d*_*i*_ is a diffusion coefficient of the JAK/STAT signaling, *k*_*i*_ is a degradation rate, and *J*_0_ is a positive constant corresponding to the JAK/STAT activation in the lateral region. From the first equation of eq. 3, the activity of JAK/STAT signaling is changed by the diffusion and the decay. We impose the zero flux boundary condition in the second formula similar to the eq. 1^13^. This indicates that the JAK/STAT activity is dependent on the distance from the ligand source and it shows the same value along with the *y* axis. In the third formula, we set activation of JAK/STAT as 0 at the medial side and *J*_0_ at the lateral side, since JAK/STAT activation was not found in the medial most region in the optic lobe and the activation in the lateral side was always strong (Fig. S1). We designate the improved version from eq. 2 as Model 2. We next performed the numerical simulations with Model 2 in a two dimensional field, *B* (Fig. 3). Even in the presence of a small perturbation in the initial condition and in each step of all variables, the proneural wave propagates similarly to the noise-free condition because of the cancelation of the noise. This is explained from the perspective of mathematical modeling: In Model 2, the term inhibiting the differentiation of *A* is reproduced by the function max{*E*_*i*,*j*_  − *N*_*i*, *j*_ − *k*_*in*_*J*_∞_, 0}. If the concentration of *E*_*i*,*j*_, which can promote the propagation of the proneural wave, is larger than that of *N* + *k*_*in*_*J*_∞_, which inhibits the propagation of the proneural wave in a cell, differentiation starts in that cell. On the other hand, *N* is not upregulated in the region distant from the wavefront^12^. Thus, stimulation of a small amount of noise to *E* eventually causes spontaneous NB differentiation. However, as the JAK/STAT signaling positively regulates Notch targets, the value inside the max function remains negative, even if a small perturbation is added into the concentration of *E* away from the wavefront. Thus, the spontaneous differentiation in the region distant from the wavefront is inhibited by the cancelation of the noise resulting from the function *J*_∞_. As *E* is the only variable that increases the value inside the max function of *A*, total noise from other components can be integrated in *E*. Thus, we can determine that the noise can be canceled if the level of the noise of *E* is less than that of *k*_*in*_*J*_∞_. The revised mathematical model still mimics *in vivo* situations and shows the capacity for noise resistance, suggesting that JAK/STAT signaling contributes to noise canceling during the progression of the proneural wave *in silico*.

In the current model, the noise resistance is less prominent when the position *x* is near the origin (Fig. 3). However, *in vivo*, the NE cells are dividing rapidly. Therefore, the undifferentiated region ahead of the wavefront is initially much smaller but is expanded as the wave propagates^8^. A future model that integrates the NE cell division will resolve this inconsistency.

### JAK/STAT suppresses ectopic NB differentiation *in vivo*

**Figure 4:**
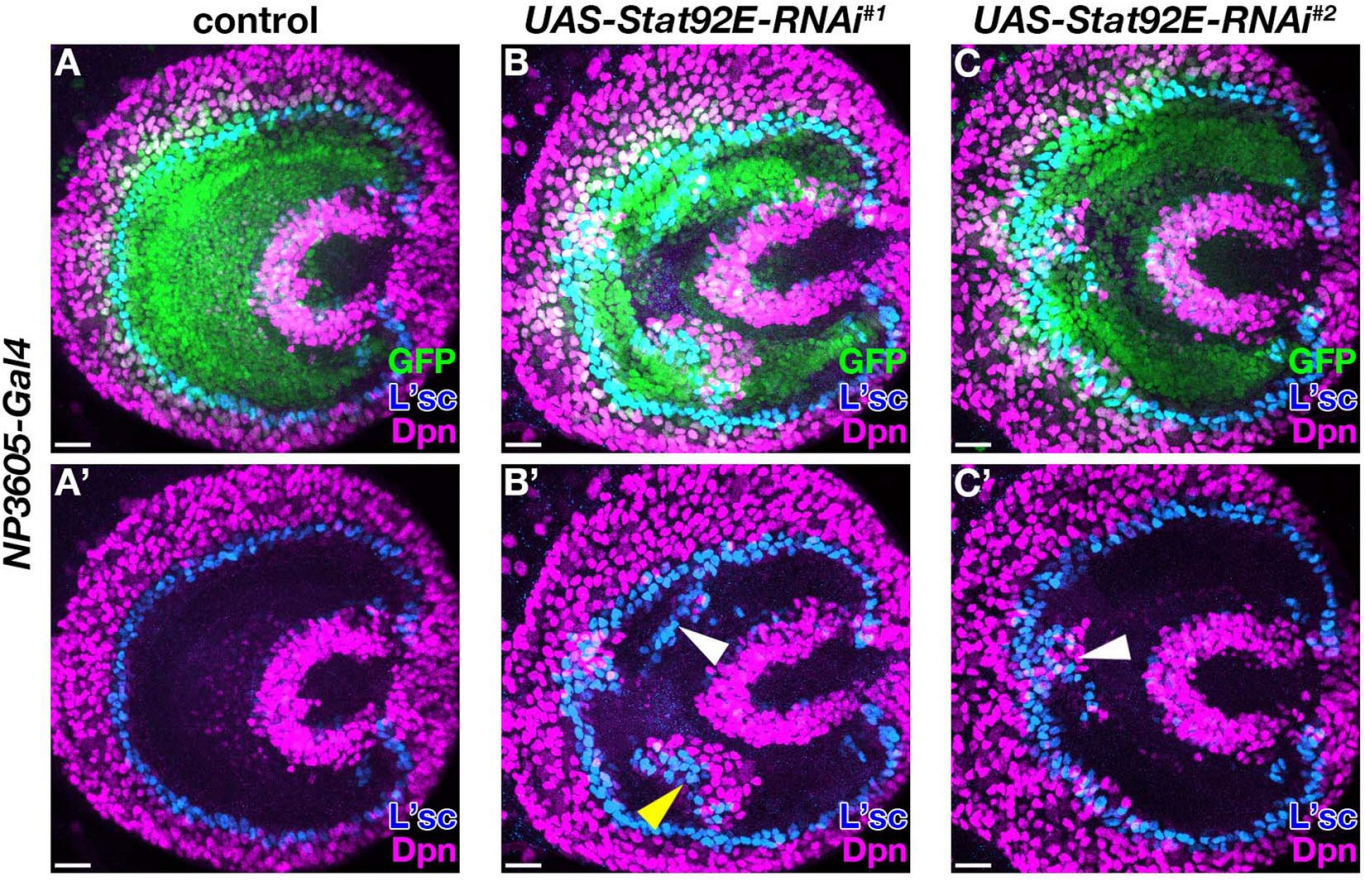
*Stat92E* blocks spontaneous neuroblast differentiation *in vivo*. (**A**–**C’**) Third-instar larval optic lobes for control (**A**), *UAS-Stat92E-RNAi*^*#1*^ (**B**), and *UAS-Stat92E-RNAi*^*#2*^ (**C**). *NP3605-Gal4 UAS-GFP*.*nls* (green), L’sc (blue) and Dpn (magenta) are shown. (**A’**–**C’**) L’sc and Dpn are shown. White arrowheads in (**B’**,**C’**) indicate ectopic L’sc and Dpn expression. The yellow arrowhead indicates ectopic L’sc expression medial to ectopic Dpn expression. Scale bars, 20 μm.

Next, to confirm the possibility that JAK/STAT cancels spontaneous NB differentiation caused by biological noise *in vivo*, we reduced the JAK/STAT signaling activity by knocking down *Stat9*2*E*. We induced *Stat9*2*E* RNAi by using the *NP3605-Gal4* driver in the larval optic lobe NEs^12^. Two *Stat9*2*E* RNAi lines targeting different regions of the *Stat92E* gene showed ectopic L’sc-positive differentiating cells and Deadpan (Dpn)-positive NBs in a stochastic manner (Fig. 4) (n = 16/18 for *UAS-Stat92E-RNAi*^*#1*^ and n = 18/44 for *UAS-Stat92E-RNAi*^*#2*^), suggesting that JAK/STAT does indeed cancel the biological noise. Additionally, the numerical simulation shows that the addition of noise away from the wavefront causes spontaneous NB differentiation in the opposite direction from that of the original proneural wave (Fig. 2). This numerical result was experimentally reproduced *in vivo*. Ectopic L’sc-expressing cells were sometimes found in the medial region, adjacent to ectopic Dpn-expressing cells, demonstrating that the ectopic NB differentiation wave progressed from the lateral to the medial direction in *STAT92E* RNAi samples (Fig. 4B’, yellow arrowhead). To exclude the possibility that the effect of RNAi was not uniform and ectopic differentiation was caused where the JAK/STAT activity was particularly reduced by *Stat92E* RNAi, we examined the activation level of JAK/STAT signaling. 10×STAT-GFP, a JAK/STAT responsive reporter, was strongly expressed in the lamina and weakly in NEs in wild type optic lobes, as previously described (Fig. S1A)^8,15^. Expression of 10xSTAT-GFP was uniformly decreased in *Stat92E* RNAi samples (Fig. S1B and S1C) (n = 31/31 for *UAS-Stat92E-RNAi*^*#1*^ and n = 84/84 for *UAS-Stat92E-RNAi*^*#2*^). These experimental results suggest that JAK/STAT signaling is required *in vivo* for preventing spontaneous NB differentiation by suppressing the effects of noise.

### Noise canceling function by JAK/STAT is mediated by EGF and Notch signaling pathways

**Figure 5:**
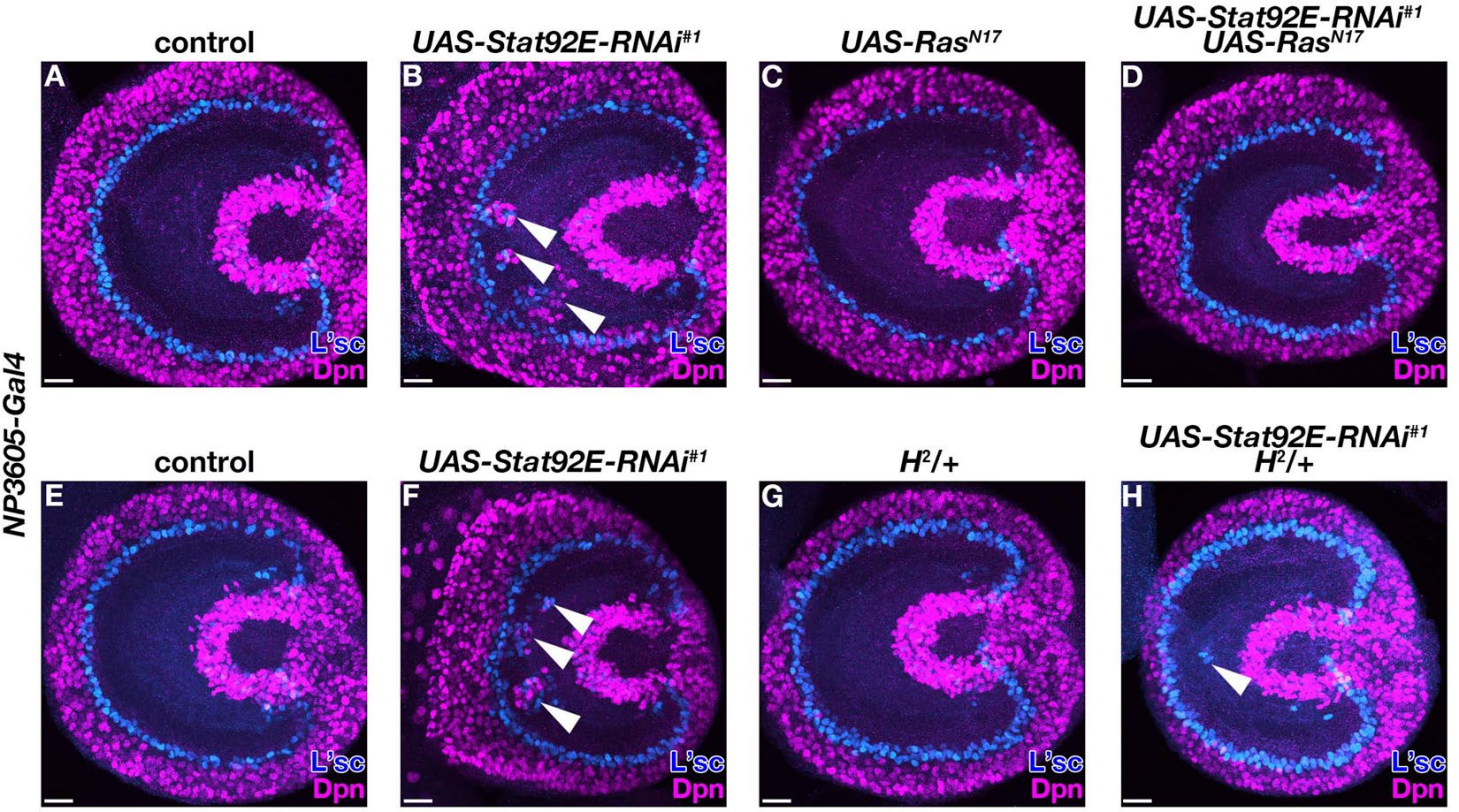
Noise canceling function of JAK/STAT is mediated by EGF and Notch signaling pathways. (**A**–**D**) Third-instar larval optic lobes for control (**A**), *UAS-Stat92E-RNAi*^*#1*^ (**B**), *UAS-Ras*^*N17*^ (**C**) and *UAS-Stat92E-RNAi*^*#1*^, *UAS-Ras*^*N17*^ (**D**). (**E**–**H**) Third-instar larval optic lobes for control (**E**), *UAS-Stat92E-RNAi*^*#1*^ (**F**), *H*^*2*^ heterozygous mutant (**G**) and *UAS-Stat92E-RNAi*^*#1*^ in the *H*^*2*^ heterozygous mutant background (**H**). *NP3605-Gal4* was used as a *Gal4* driver. L’sc (blue) and Dpn (magenta) are shown. White arrowheads in (**B**,**F**,**H**) indicate ectopic L’sc and/or Dpn expression. Scale bars, 20 μm.

In order to further dissect the noise canceling mechanism by JAK/STAT, we first asked whether activation of EGF signaling is required for ectopic NB differentiation under the *Stat92E* RNAi background. To reduce EGF activation, we expressed Ras^N17^, a dominant negative form of Ras (Fig. 5A–D)^16^. Ras^N17^ expression in the control background did not show any phenotype (Fig. 5C) (n = 0/36). The phenotype of ectopic L’sc or Dpn expression by *Stat92E* RNAi (Fig. 5B) (n = 15/15) was suppressed by simultaneous expression of Ras^N17^ (Fig. 5D) (n = 0/58). This result suggests that EGF activation can be a source of noise which induces spontaneous NB differentiation under the decreased JAK/STAT activation condition.

We next examined the possibility that Notch signaling is involved in noise canceling. To test whether expression of *E(spl)mδ* and *E(spl)m7* is altered under the *Stat92E* RNAi condition, we checked expression pattern of E(spl)mδ-GFP and E(spl)m7-GFP. E(spl)mδ-GFP was expressed in cells in the lamina and in wavefront cells (Fig. S2A). E(spl)mδ-GFP expression in the lamina was disturbed when ectopic NB differentiation was induced by *Stat92E* RNAi (Fig. S2B) (n = 24/32). E(spl)m7-GFP was expressed in the lamina and NEs including wavefront cells (Fig. S2C). E(spl)m7-GFP was partially lost upon Stat92E knockdown (arrowhead in Fig. S2D) (n = 6/32). In both cases, GFP expression was only partially decreased, though *Stat92E* RNAi reduced the JAK/STAT activity uniformly (Fig. S1). Therefore, it is possible that JAK/STAT signaling regulates expression of these Notch target genes indirectly. To examine whether Notch signaling is involved in JAK/STAT-mediated noise canceling, we tested genetic interaction between the two signaling pathways (Fig. 5E–H). Hairless (H) acts as an antagonist to Notch signaling^17,18^. The ectopic NB differentiation phenotype by *Stat92E* RNAi (Fig. 5F) (n = 32/39) was partially suppressed in the *H*^*2*^ heterozygous background (Fig. 5G,H) (n = 1/50 for 5 G and n = 13/45 for 5 H). This result suggests that Notch signaling acts downstream of JAK/STAT signaling to mediate the noise canceling function, though we cannot rule out the possibility that Notch signaling acts in parallel with JAK/STAT signaling (red arrows in Fig. 1D). Importantly, however, these two alternative possibilities are equivalent in our mathematical model (eq. 2).

## Discussion

Although cellular events contain stochastic fluctuations, developmental processes progress precisely, and pattern formation is spatiotemporally organized, suggesting that there are regulatory mechanisms filtering the noise. In this report, we demonstrate that JAK/STAT signaling has a noise-canceling function during neural stem cell differentiation in the *Drosophila* optic lobe. From our mathematical model, any molecule which shows broad expression in NEs and negatively regulates EGF activation or expression of AS-C can cancel noise. However, we assume that JAK/STAT signaling plays the major role on noise canceling because JAK/STAT signaling is the only known factor which fits to the above assumptions and because the decrease of the JAK/STAT signal activation did show the ectopic neural stem cell differentiation phenotype^8^. JAK/STAT is involved in many developmental contexts and stem cell maintenance in both vertebrates and invertebrates^19–22^. Recent *in silico* experiments predict that Stat3 is involved in noise processing in mouse embryonic stem cells^23^. It is tempting to speculate that the function of JAK/STAT in noise canceling is an evolutionally conserved fundamental mechanism to regulate the robustness of stem cell maintenance and differentiation.

Recently, a complementary strategy using both mathematical modeling and *in vivo* experiments has emerged as a powerful way to elucidate multiple types of biological noise within developmental systems^5,24^. By taking advantage of mathematical modeling, we can reveal how multicellular organisms cope with intrinsic and extrinsic noise to develop a robust system. Henceforth, by applying a combination of mathematical modeling and molecular genetics, we can solve biological questions that have previously been difficult to address.

## Methods

### Mathematical modeling

In the mathematical model, the calculation region is a two dimensional field *B* = [0, *L*_*x*_] × [0, *L*_*y*_] for positive constants *L*_*x*_, *L*_*y*_ > 0 with the zero flux boundary condition. The field *B* is divided into square or hexagonal meshes in the numerical calculations, and each mesh is regarded as a cell with the indexes *i* and *j* corresponding to horizontal and vertical directions, respectively. Further, the variables without the indexes of *i* and *j* in the first equation of Model 1 indicate the variable for the whole region by combining each cell in the field *B*, and *E*_*i*, *j*_ reproduces the averaging amount of *E* in the *i*, *j*th cell. Please refer the previous study^13^ for the detailed mathematical settings.

### Fly Genetics

Flies were grown at 25 °C, and *w* or *y w* flies were used as controls. *NP3605-Gal4 UAS-GFP*.*nls*, *UAS-Stat92E-RNAi*^*#1*^ (VDRC: 43866), *UAS-Stat92E-RNAi*^*#2*^ (VDRC: 106980), *UAS-Ras*^*N17*^ (BDSC: 4845), *10xSTAT-GFP* (BDSC: 26197), *E(spl)m7-GFP* (BDSC: 55839), *E(spl)mδ-GFP* (BDSC: 68191), and *H*^*2*^ (BDSC: 517) flies were used.

### Histology

Third-instar wandering larvae were dissected in PBS and fixed in 4% formaldehyde in PBS. Samples were washed three times after fixation with PBS containing 0.3% Triton X-100 and transferred to blocking solution (PBS containing 5% normal donkey serum and 0.3% Triton X-100). Specimens were incubated overnight at 4 °C with primary antibodies diluted in blocking solution. Primary antibodies were washed four times with PBS containing 0.3% Triton X-100 before the incubation overnight at 4 °C with secondary antibodies. Then, the samples were washed four times with PBS containing 0.3% Triton X-100. The primary antibodies used were as follows: rabbit anti-PatJ (1:1000), rat anti-Dpn (Abcam, 1:50), and guinea pig anti-L’sc (1:1200). The following secondary antibodies (Jackson) were used at 1:200 dilutions: FITC-conjugated donkey anti-rabbit, Cy3-conjugated donkey anti-rat, and Alexa Fluor 647-conjugated donkey anti-guinea pig. Specimens were mounted with VectaShield mounting media (Vector) and viewed on a Zeiss LSM880 confocal microscope. ZEN software (Zeiss) was used for preparing three-dimensional images.

## Electronic supplementary material

Supplementary Dataset

## Data Availability

Data presented in this manuscript are tabulated in this published article and its Supplemental Information files.
