# Supplementary material for: JAK/STAT guarantees robust neural stem cell differentiation by shutting off biological noise
Source: Sci Rep. 2018 Aug 20;8:12484. doi: 10.1038/s41598-018-30929-1 (PMC6102247; doi:10.1038/s41598-018-30929-1)
Supplement: Supplementary file 1 — Supplementary Dataset [file 41598_2018_30929_MOESM1_ESM.pdf]

JAK/STAT guarantees robust neural stem cell differentiation by shutting off biological noise

Authors

Yoshitaro Tanaka, Tetsuo Yasugi, Masaharu Nagayama, Makoto Sato, and Shin-Ichiro Ei

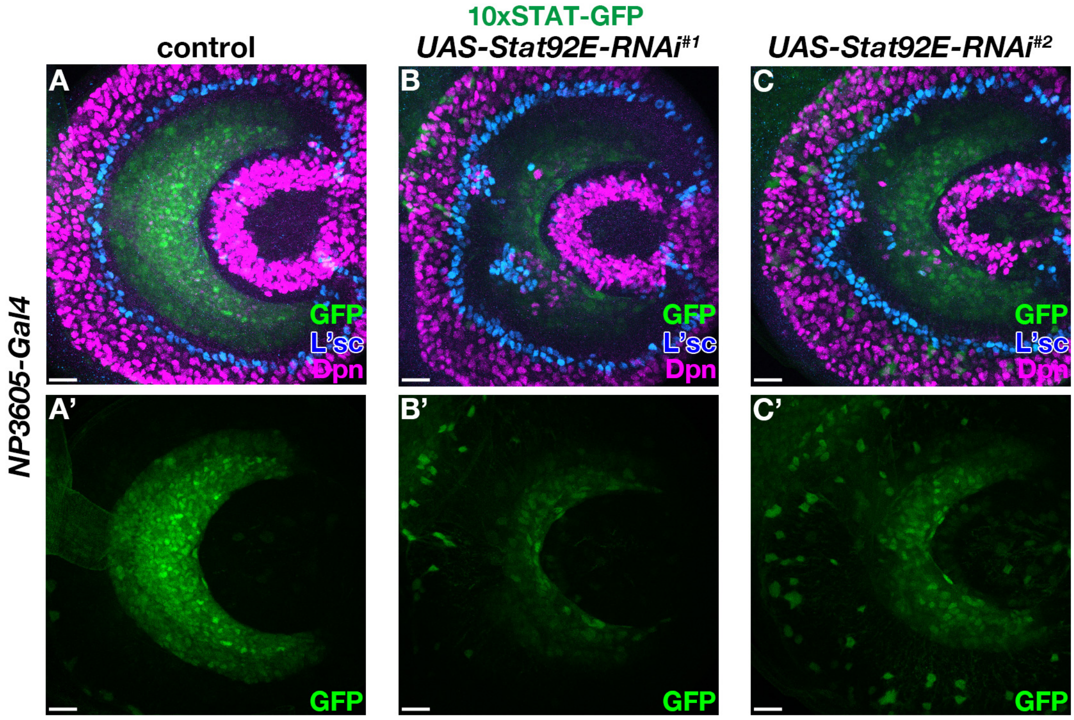

Figure S1. ***Stat92E*** **RNAi reduces the activity of JAK/STAT signaling uniformly.** (A-C') Third-instar larval optic lobes for control (A), *UAS-Stat92E-RNAi*#1 (B), and *UAS-Stat92E-RNAi*#2 (C). *NP3605-Gal4* was used as a *Gal4* driver. 10xSTAT-GFP (green), L'sc (blue) and Dpn (magenta) are shown. (A', B', and C') 10xSTAT-GFP is shown. Scale bars, 20 μm.

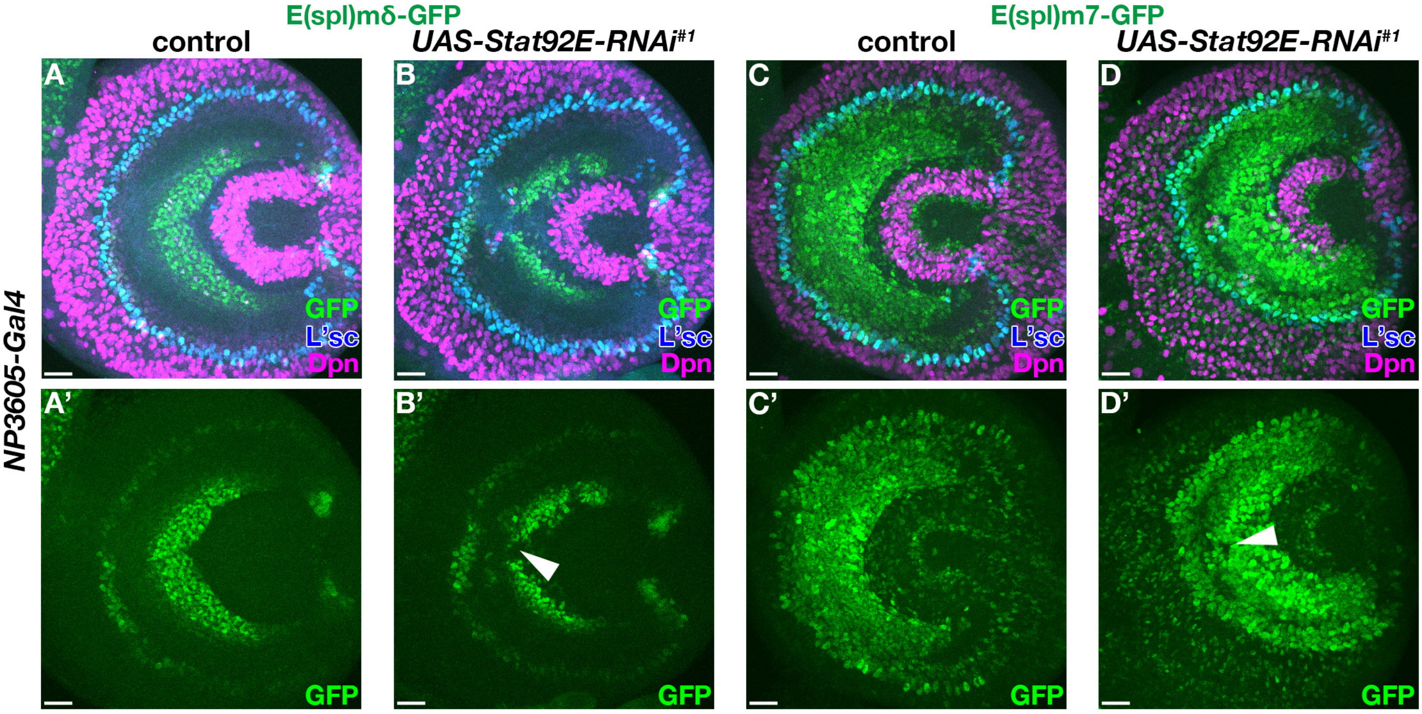

Figure S2. ***Stat92E*** **RNAi reduces the activity of JAK/STAT signaling uniformly.** (A-B') Third-instar larval optic lobes for control (A) and *UAS-Stat92E-RNAi*[#1] (B). E(spl)mδ-GFP (green), L'sc (blue) and Dpn (magenta) are shown. (A' and B') E(spl)mδ-GFP is shown. (C-D') Third-instar larval optic lobes for control (C) and *UAS-Stat92E-RNAi*[#1] (D). E(spl)m7-GFP (green), L'sc (blue) and Dpn (magenta) are shown. (C' and D') E(spl)m7-GFP is shown. *NP3605-Gal4* was used as a *Gal4* driver. White arrowheads in (B') and (D') indicate the decrease of GFP expression. Scale bars, 20 μm.
